# Rescue Therapy for *Helicobacter pylori* Infection 2012

**DOI:** 10.1155/2012/974594

**Published:** 2012-02-28

**Authors:** Javier P. Gisbert

**Affiliations:** Department of Gastroenterology, Hospital Universitario de La Princesa, Instituto de Investigación Sanitaria Princesa (IP), and Centro de Investigación Biomédica en Red de Enfermedades Hepáticas y Digestivas (CIBEREHD), 28006 Madrid, Spain

## Abstract

*Helicobacter pylori* infection is the main cause of gastritis, gastroduodenal ulcer disease, and gastric cancer. After 30 years of experience in *H. pylori* treatment, however, the ideal regimen to treat this infection has still to be found. Nowadays, apart from having to know well first-line eradication regimens, we must also be prepared to face treatment failures. In designing a treatment strategy, we should not only focus on the results of primary therapy alone but also on the final—overall—eradication rate. The choice of a “rescue” treatment depends on which treatment is used initially. If a first-line clarithromycin-based regimen was used, a second-line metronidazole-based treatment (quadruple therapy) may be used afterwards, and then a levofloxacin-based combination would be a third-line “rescue” option. Alternatively, it has recently been suggested that levofloxacin-based “rescue” therapy constitutes an encouraging 2nd-line strategy, representing an alternative to quadruple therapy in patients with previous PPI-clarithromycin-amoxicillin failure, with the advantage of efficacy, simplicity and safety. In this case, quadruple regimen may be reserved as a 3rd-line “rescue” option. Even after two consecutive failures, several studies have demonstrated that *H. pylori* eradication can finally be achieved in almost all patients if several “rescue” therapies are consecutively given.

## 1. Introduction

*Helicobacter pylori* infection is the main known cause of gastritis, gastroduodenal ulcer disease, and gastric cancer. After 30 years of experience in *H. pylori* treatment, however, the ideal regimen to treat this infection has still to be found [1–3]. Consensus conferences have recommended therapeutic regimens that achieve *H. pylori* cure rates higher than 80% on an intention-to-treat basis [4–7]. However, several large clinical trials and meta-analyses have shown that the most commonly used first-line therapies, including proton pump inhibitors (PPIs) plus two antibiotics, may fail in ≥20% of patients [8, 9], and in the clinical routine setting, the treatment failure rate might be even higher [10, 11]. Moreover, during the last few years, the efficacy of PPI-based regimens seems to be decreasing, and several studies have reported intention-to-treat eradication rates lower than 75% and even lower than 50% [12–15]. Antibiotic resistance to clarithromycin has been identified as one of the major factors affecting our ability to cure *H. pylori* infection, and the rate of resistance to this antibiotic seems to be increasing in many geographical areas [16–19].

Several “rescue” therapies have been recommended, but they still fail to eradicate *H. pylori* in more than 20% of the cases, and these patients constitute a therapeutic dilemma [20–22]. Patients who are not cured with two consecutive treatments including clarithromycin and metronidazole will have at least single, and usually double, resistance [17, 23]. Furthermore, bismuth salts are not available worldwide anymore; therefore, management of first-line eradication failures is becoming challenging. Currently, a standard third-line therapy is lacking, and European guidelines recommend culture in these patients to select a third-line treatment according to microbial sensitivity to antibiotics [5, 6]. However, cultures are often carried out only in research centers, and the use of this procedure as “routine practice” in patients who failed several treatments seems not to be feasible [20, 21, 24–26]. Therefore, the evaluation of drugs without cross-resistance to nitroimidazole or macrolides as components of retreatment combination therapies would be worthwhile [27, 28].

All these issues are important at the present time, but they will be even more relevant in the near future, as therapy for *H. pylori* infection is becoming more and more frequently prescribed. Therefore, the evaluation of second or third “rescue” regimens for these problematic cases seems to be worthwhile [29]. In designing a treatment strategy, we should not focus on the results of primary therapy alone; an adequate strategy for treating this infection should use several therapies which, if consecutively prescribed, come as close to the 100% cure rate as possible [20, 21, 25, 26, 30, 31].

The aim of the present paper will be to review the experience dealing with “nonresponders” to *H. pylori* eradication therapy, and specifically with *H. pylori* “rescue” therapies after failure of the first-line eradication regimen. As, at present, the current most prescribed first-line regimens include a combination of PPI plus two antibiotics, the present paper will focus on “rescue” regimen when these triple combinations fail. Bibliographical searches were performed in the PubMed (Internet) database including studies available until October 2011, looking for the following words (all fields): pylori AND (retreatment OR re-treatment OR rescue OR failure OR salvage OR second-line).

## 2. Is It Necessary to Perform Culture After Failure of the First Eradication Treatment?

Pretreatment antibiotic resistance is the most important factor in nonresponse to initial treatment [32]. Thus, the choice of a second-line treatment depends on which treatment was used initially, as it would appear that retreatment with the same regimen cannot be recommended [33]. If a clarithromycin-based regimen was used, a metronidazole-based treatment (or at least a clarithromycin-free regimen) should be used afterwards, and *vice versa* [34]. This recommendation is based on the observation that acquired bacterial resistance to metronidazole or clarithromycin results primarily from the previous treatment failure [32], and therefore “rescue” therapies should avoid these antibiotics and use different combinations.

An antimicrobial susceptibility test for *H. pylori* before second-line treatment is sometimes performed, although whether the test is truly necessary remains unknown. Some authors have evaluated the efficacy of susceptibility-guided versus empiric retreatment for *H. pylori* after a treatment failure. In the study by Yahav et al. [35], patients in whom at least one treatment regimen for *H. pylori* eradication had failed underwent gastric biopsy and culture and were retreated according to the *in vitro* susceptibility results. Findings were compared with those for control patients (where culture was unavailable). Susceptibility-guided retreatment was associated with better eradication rates (86%) than empiric treatment (63%). However, several methodological drawbacks exist in this study. Firstly, more than 50% of the patients received first-line eradication treatment with both clarithromycin and metronidazole (instead of including clarithromycin and amoxicillin), which is not the generally recommended combination; consequently, no logical empirical treatment remained afterwards (levofloxacin-based regimens were not available at that time). In this respect, when only the eradication rates in control (culture unavailable) patients treated with a first regimen of PPI-amoxicillin-clarithromycin followed by a second *empiric* quadruple regimen were considered (the generally recommended first- and second-line strategies), the success figures were not significantly different from those reported in patients receiving susceptibility-guided retreatment. Secondly, because this study was nonrandomized, there might have been heterogeneity among the two groups with respect to the treatment regimens prescribed by the treating physicians. Finally, this study was limited by the lack of susceptibility data for the controls, which restricted the ability to analyze the reasons why empiric therapy did not work as well as the susceptibility-guided protocol.

In a French multicenter study [36], patients, in whom one previous *H. pylori* eradication therapy (mainly with PPI-amoxicillin-clarithromycin) has failed, were randomized to receive one of three empirical triple-therapy regimens or a strategy based on antibiotic susceptibility. The empirical regimens were PPI-amoxicillin-clarithromycin (for 7 or 14 days) or PPI-amoxicillin-metronidazole (for 14 days). In the susceptibility-based strategy, patients with clarithromycin-susceptible strains received PPI-amoxicillin-clarithromycin, whilst the others received PPI-amoxicillin-metronidazole. The eradication rates for empirical therapies were low, while the cure rate was higher (74%) for the susceptibility-based treatment. If the *H. pylori* strain was clarithromycin-susceptible (which occurred in approximately 1/3 of the cases), a high-success rate was obtained with the PPI-clarithromycin-amoxicillin “rescue” regimen. The study, however, was done in France, where bismuth is banned, so that the use of quadruple therapy with a PPI, bismuth, tetracycline, and metronidazole as recommended by the updated Maastricht Consensus Report [6], was not tested. In fact, as it will be reviewed later, several studies have obtained relatively good results with this quadruple regimen empirically prescribed, with mean eradication rate of 77% (i.e., a similar figure than the 74% achieved for the susceptibility-based treatment in the present study). Thus, in this study, instead of not readministering any of the antibiotics against which *H. pylori* has probably become resistant, the authors insist on prescribing again clarithromycin (or metronidazole) for the second-line treatment. Furthermore, statistically significant differences were not demonstrated when comparing the efficacy of the empirical PPI-amoxicillin-metronidazole and the susceptibility-based strategy, suggesting that the metronidazole-based combination may be an effective empirical alternative after failure of a clarithromycin-based combination.

In the updated Maastricht Consensus Report [6], it was recommended that culture and antimicrobial sensitivity testing should be routinely performed only after two treatment failures with different antibiotics. According to this statement, some studies have suggested that an antimicrobial susceptibility test for *H. pylori* before administering second-line treatment is not necessary. In this respect, in the study by Avidan et al. [37], after failure of first-line eradication treatment, half of the patients were randomly assigned to treatment with a different PPI-based triple regimen regardless of the culture obtained, and the other half were assigned to treatment with PPI and two antibacterial agents chosen according to a susceptibility test; the authors found that the culture results did not influence the treatment protocol employed. Similarly, in the study by Miwa et al [38], patients with *H. pylori* infection for whom first-line treatment with a PPI-amoxicillin-clarithromycin regimen had failed were randomly assigned to two groups: those having or not having the susceptibility test before retreatment. For those patients in the susceptibility-test group, the authors used what they considered the best regimen based on susceptibility testing; while for those patients in the group with no susceptibility testing, PPI-amoxicillin-metronidazole was prescribed. The cure rates in the groups with and without susceptibility testing were not different.

## 3. Second-Line *H. pylori* “Rescue” Therapy after Failure of One Eradication Treatment

### 3.1. “Rescue” Regimen after PPI-Clarithromycin-Amoxicillin Failure

#### 3.1.1. PPI, Amoxicillin, and Metronidazole

After failure of a combination of PPI, amoxicillin, and clarithromycin, a theoretically correct alternative would be the use, as second option, of other PPI-based triple therapy including amoxicillin (that does not induce resistance) and metronidazole (an antibiotic not used in the first trial), and several authors have reported encouraging results with this strategy [38–45]. However, in our experience, when this therapy has been administered twice daily for one week, eradication rates lower than 50% have been obtained [46]; the subsequently use of higher (three times per day) antibiotic doses was followed only by a mild increase in eradication rate (58%), which was still unacceptable [46]. Nagahara et al. [47] studied a group of patients who, after failure of first-line PPI-clarithromycin-amoxicillin therapy, had received second-line therapy with the same regimen (for 14 days) or had received PPI-amoxicillin-metronidazole (for 10 days). The eradication rate for second-line therapy with the same regimen (thus readministering clarithromycin) was of only 53%, while it was of 81% with PPI-amoxicillin-metronidazole. These observations underlie the idea that antibiotics, and specifically clarithromycin, should not be readministered in successive treatments.

#### 3.1.2. Quadruple Therapy

Another alternative, the use of a quadruple regimen (i.e., PPI, bismuth, tetracycline, and metronidazole), has been generally used as the optimal second-line therapy after PPI-clarithromycin-amoxicillin failure and has been the recommended “rescue” regimen in several guidelines [6, 48, 49]. Several studies have obtained relatively good results with this quadruple regimen, the results are summarized in Table 1 [46, 50–64]. Thus, the weighted mean eradication rate with this “rescue” therapy, calculated from the studies included in the table, is of 77%. In this combination regimen, PPI should be prescribed in the usual dose and twice a day, colloidal bismuth subcitrate 120 mg four times per day, tetracycline 500 mg four times per day, and metronidazole is probably best prescribed at high doses (i.e., 500 mg three times per day). Precisely, the study with the lowest efficacy [56] administered metronidazole at low doses (250 mg four times per day). Although PPIs are generally prescribed as the antisecretors in quadruple therapy, some authors have shown, in a randomized study, that omeprazole 20 mg b.i.d. and ranitidine 300 mg b.i.d. were equally effective as antisecretory agents combined in a second-line quadruple eradication regimen after failure with previous regimens without metronidazole (although the power of the study to find statistically significant differences was limited) [61]. Nevertheless, these regimens were administered during 14 days; therefore, it remains to be demonstrated if the equivalence between both antisecretors—PPIs and H_2_-blockers—is also observable with 7-day or 10-day regimens.

The question may be suggested whether treatment with PPI-clarithromycin-amoxicillin followed by “rescue” with quadruple therapy if failure is preferable to the inverse strategy. To analyze this interesting aspect, Gomollón et al. [65] randomized consecutive patients to one of two strategies: (a) treatment during 7 days with quadruple therapy, and if failure second-line treatment with omeprazole-clarithromycin-amoxicillin during 7 days, (b) initial treatment with omeprazole-clarithromycin-amoxicillin, and if failure treatment with quadruple therapy. Direct and indirect costs were estimated, and a cost-effectiveness analysis using a decision-tree model was undertaken after real clinical data. Eradication was obtained (intention-to-treat) in 73% with the first strategy, *versus* 92% with the second one. Furthermore, cost per case eradicated was lower in the second group (320 *versus* 296 Euros). However, in a similar but more recent study, Marko et al. [60] assessed the usefulness and the cost-effectiveness of these two treatment strategies, performing a decision analysis. The effectiveness of “triple first” and “quadruple first” strategies was similar, although the latter seemed slightly more cost-effective.

#### 3.1.3. PPI, Amoxicillin, and Levofloxacin

As previously mentioned, after failure of a combination of a PPI-based triple regimen, the use of the quadruple therapy has been generally recommended as the optimal second-line therapy based on the relatively good results reported by several authors. However, this quadruple regimen requires the administration of 4 drugs with a complex scheme (bismuth and tetracycline usually prescribed every 6 hours, and metronidazole every 8 hours) and is associated with a relatively high incidence of adverse effects [20]; however, this drawback may be overcome, thanks to a novel single capsule containing bismuth, metronidazole, and tetracycline that has recently become available [66, 67]. Nevertheless, this quadruple regimen still fails to eradicate *H. pylori* in approximately 20 to 30% of the patients, and these cases constitute a therapeutic dilemma, as patients who are not cured with two consecutive treatments including clarithromycin and metronidazole will usually have double resistance [20].

Levofloxacin is a fluoroquinolone antibacterial agent with a broad spectrum of activity against Gram-positive and Gram-negative bacteria and atypical respiratory pathogens [68]. Recently, some studies have evaluated the efficacy of new fluoroquinolones, such as levofloxacin, that could prove to be a valid alternative to standard antibiotics not only as first-line therapies but, more interesting, as second-line regimens [21, 69–71]. In this respect, levofloxacin-based second-line therapies represent an encouraging strategy for eradication failures, as some studies have demonstrated that levofloxacin has, *in vitro*, remarkable activity against *H. pylori* [72], and that primary resistances to such antibiotic in several countries are (still) relatively infrequent (when compared with metronidazole or clarithromycin) [73–80]. A recent *in vitro* study also showed a synergistic effect of quinolone antimicrobial agents and PPIs on strains of *H. pylori* [81]. Furthermore, it has been shown *in vitro* that levofloxacin retains its activity when *H. pylori* strains are resistant to clarithromycin and metronidazole [76, 82, 83]. These favorable results have been confirmed *in vivo*, indicating that most of the patients with both metronidazole and clarithromycin resistance are cured with the levofloxacin-based regimen [51, 75, 84, 85].

A combination of a PPI, amoxicillin and levofloxacin, as first-line regimen, has been associated with favorable results, with mean eradication rates of about 90% [76, 86–91]. Later, other authors studied this same regimen in patients with one previous eradication failure, also reporting exciting results, with *H. pylori* cures rates ranging from 60% to 94%% [51, 63, 82, 84, 91–104]. A recent systematic paper showed a mean eradication rate with levofloxacin-based “rescue” regimens (combined with amoxicillin and a PPI in most studies) of 80%, which represents a relatively high figure when considering that this regimen was evaluated as a “rescue” therapy [70]. This systematic paper found, in agreement with recent randomized clinical trials [105], higher *H. pylori* cure rates with 10-day than with 7-day regimens, both in general (81% versus 73%) and also with the levofloxacin-amoxicillin-PPI combination in particular (80% versus 68%), suggesting that the longer (10-day) therapeutic scheme should be chosen.

**Figure 1:**
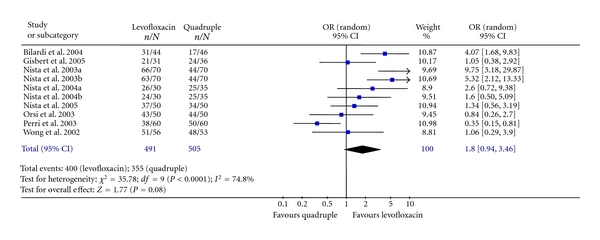
Meta-analysis comparing *H. pylori* eradication efficacy with levofloxacin-based triple regimens *versus* quadruple therapy, as second-line “rescue” regimen after failure of a proton pump inhibitor-amoxicillin-clarithromycin.

Furthermore, three recent meta-analyses have suggested that after *H. pylori* eradication failure, levofloxacin-based “rescue” regimen is more effective than the generally recommended quadruple therapy [69, 70, 106]. In one of these meta-analyses [70], higher *H. pylori* cure rates with the levofloxacin-based triple regimens than with the quadruple combinations were found (81% versus 70%), but with borderline statistical significance (Figure 1). Nevertheless, results were heterogeneous, mainly due to the discordant results of the study by Perri et al. [92], who reported a cure rate of only 63% with the levofloxacin-regimen, the lowest reported in the literature, a figure that contrasts with the mean eradication rate of 80% calculated in a systematic paper [70]. Nevertheless, when that single outlier study [92] was excluded from the meta-analysis, the difference between cure rates with both regimens reached statistical significance and heterogeneity markedly decreased. Furthermore, when only high-quality studies were considered, the advantage of the levofloxacin regimen over the quadruple regimen increased (88% versus 64%), also achieving statistical significance, and heterogeneity among studies almost disappeared [70]. Nevertheless, the benefit of the levofloxacin-based “rescue” regimen seems to be less clear in Asia, as two studies from Taiwan and Hong Kong showed that levofloxacin-based triple therapies were at most comparable to quadruple therapy [95, 102].

As previously mentioned, the quadruple regimen requires the administration of a complex scheme [20]. On the contrary, levofloxacin-based regimens (with amoxicillin and PPIs administered twice daily, and levofloxacin every 12 or 24 hours) represent an encouraging alternative to quadruple therapy, with the advantage of simplicity. Furthermore, the quadruple regimen is associated with a relatively high incidence of adverse effects [20]. In contrast, levofloxacin is generally well tolerated, and most adverse events associated with its use are mild to moderate in severity and transient [68]. The most frequent adverse effects affect the gastrointestinal tract [68]. Occasional cases of tendinitis and tendon rupture have been reported in the literature with levofloxacin therapy [51, 68]. However, data derived from more than 15 million prescriptions in the US indicated the rate is fewer than 4 per million prescriptions [107]. *Clostridium difficile* infection may be associated with the use of this broad spectrum activity antibiotic [68]. In the aforementioned systematic review [70], adverse effects were reported, overall, by 18% of the patients treated with levofloxacin-based therapies, and these adverse effects were severe (defined so by the authors or explaining treatment discontinuation) in only 3% of the cases. Furthermore, the incidence of adverse effects was not different when levofloxacin-amoxicillin-PPI was administered for 7 or 10 days, supporting the aforementioned recommendation of prescribing the more effective 10-day regimen. Moreover, two meta-analyses have demonstrated a lower incidence of adverse effects with levofloxacin-based treatments than with the quadruple combinations [69, 70]. Finally, it has recently been demonstrated that moxifloxacin-containing triple regimen is more effective and better tolerated than the bismuth-containing quadruple therapy in the second-line treatment of *H. pylori* infection [106].

Unfortunately, it has been shown that resistance to quinolones is easily acquired, and in countries with a high consumption of these drugs, the resistance rate is increasing and is already relatively high [75, 88, 95, 108–125]. More importantly, it has been demonstrated that the presence of levofloxacin resistance significantly reduce the eradication rate following a therapy with this antibiotic [75, 88, 118, 126, 127]. Therefore, it has been suggested to reserve levofloxacin for “rescue” treatment to avoid the increase of the resistance phenomenon [128].

### 3.2. “Rescue” Regimen after PPI-Amoxicillin-Nitroimidazole Failure

After PPI-amoxicillin-nitroimidazole failure, retreatment with PPI-amoxicillin-clarithromycin has proved to be very effective, and it seems to be a logical strategy, as while amoxicillin is maintained (which does not induce resistance), clarithromycin is substituted for metronidazole. Furthermore, the absence of cross-resistance among nitroimidazoles and clarithromycin favors this position. With this therapy, some authors [46] have achieved *H. pylori* eradication in 85% of cases, while others have reported success rates of 86% [129] or even 100%% [130]. In favor of this strategy is the study by Magaret et al. [131] who studied a group of 48 patients after failure of previous *H. pylori* therapy with a metronidazole-containing regimen and randomized them to either lansoprazole, amoxicillin, and clarithromycin twice daily for 14 days (i.e., the logical approach with triple therapy not repeating metronidazole) or to lansoprazole, bismuth, metronidazole and tetracycline for 14 days (i.e., the quadruple therapy repeating metronidazole). Intention-to-treat efficacies were 75% for triple regimen and 71% for quadruple. Although this difference did not reach statistical significance, the small sample size of this study does not preclude the possibility of a small but clinically significant difference in efficacy between the regimens.

### 3.3. “Rescue” Regimen after PPI-Clarithromycin-Nitroimidazole Failure

As previously mentioned, acquired bacterial resistance to metronidazole or clarithromycin results primarily from the previous treatment failure [132], and therefore the first choice probably should not be a regimen that combines these two antibiotics in the same regimen [30, 31, 133]. Although this regimen is very effective [8], patients who are not cured will probably have double resistance [134, 135], and no logical empirical treatment remains afterwards (although, more recently, the levofloxacin-based regimens may represent an option). Thus, some authors have demonstrated that initial regimens containing both clarithromycin and nitroimidazole are associated with significantly worse results *overall*, with lower eradication rates after logically chosen second-line therapy and sensitivity-directed third-line therapy; these poor results were due to the emergence of multiply resistant strains as evidenced by the results of culture testing after the second failed course [136]. In summary, due to problems with resistance it could be suggested that both key antibiotics—clarithromycin and metronidazole—should not be used together until a valid empirical back up regimen is available [30].

Nevertheless, if culture is not performed after failure of PPI-clarithromycin-metronidazole, and hence antibiotic susceptibility is unknown, several “rescue” options may be suggested. Firstly, omeprazole plus amoxicillin, with a high dose of both the antibiotic and the antisecretor, could, in theory, be recommended [133, 137]; however, we must remember that this “old-fashioned” dual combination has achieved disappointing results in many countries [138]. Therefore, a second antibiotic should be added, and at this point a difficult decision appears, as both antibiotics used in the first trial (clarithromycin and metronidazole) are capable of inducing secondary resistance to *H. pylori*, playing a negative role in future efficacy [134, 139–144]. Nevertheless, the following possibilities exist.

#### 3.3.1. Readministering Metronidazole

Due to the fact that metronidazole resistance is frequent and clinically relevant [134, 139–141], if this antibiotic is readministered, it should be used within bismuth-based quadruple regimen (thus PPI might reduce the negative effect of metronidazole resistance [57, 141, 145]). With this regimen, eradication rates up to 80% have been achieved [46].

#### 3.3.2. Readministering Clarithromycin

Several studies have underlined the relevance of clarithromycin resistance [134, 139, 140, 142], which advise against readministering this antibiotic. Therefore, a further option which has been proposed is to add (e.g., to PPI-amoxicillin-clarithromycin) a fourth medication (as bismuth [146, 147]) with bactericidal effect against *H. pylori*, with which 70% eradication rate has been achieved [46].

#### 3.3.3. Readministering No Antibiotic

A final alternative, obviously, consists of no readministering either metronidazole or clarithromycin. Although only published in abstract form, one study has prescribed ranitidine bismuth citrate, tetracycline, and amoxicillin for 2 weeks and has reported the eradication in 89% of the cases who had previously failed PPI, clarithromycin, and tinidazole [148]. These encouraging results may be due, at least in part, to the use of ranitidine bismuth citrate instead of bismuth in this regimen, as “classic” triple therapy with bismuth, tetracycline, and amoxicillin has been previously considered relatively ineffective. Finally, although not specifically evaluated in PPI-clarithromycin-metronidazole failures, rifabutin, or levofloxacin-based regimens (e.g., PPI, amoxicillin and either levofloxacin or rifabutin) could play a role in this difficult situation. However, several concerns remain regarding rifabutin treatment [149]. Firstly, this drug is very expensive. Secondly, severe leucopoenia and thrombocytopenia have been reported in some patients treated with rifabutin. Finally, there is some concern about a wide-spread use of rifabutin, a member of a class of established antimycobacterial drugs, in patients with *H. pylori* infection. Because multiresistant strains of *Mycobacterium tuberculosis* increase in numbers, indications for these drugs should be chosen very carefully to avoid further acceleration of development of resistance.

### 3.4. “Rescue” Regimen after Nonbismuth Quadruple “Sequential” and “Concomitant” Treatment Failure

As previously mentioned, the most widely recommended treatment for the eradication of *H. pylori* is the standard, or PPI-based triple therapy, which combines 2 antibiotics (clarithromycin plus amoxicillin or metronidazole). However, one recent innovation, postulated as an alternative to standard triple therapy, is sequential treatment, which involves a simple dual regimen including a PPI plus amoxicillin for the first 5 days followed by a triple regimen including a PPI, clarithromycin, and tinidazole for the following 5 days [2]. On the other hand, the concept of a nonbismuth quadruple regimen or “concomitant” regimen has recently resurfaced. Traditional standard triple therapy (PPI-clarithromycin-amoxicillin) can easily be converted to “concomitant” therapy by the addition of 500 mg of metronidazole or tinidazole twice daily [3].

It remains unclear how failure of non-bismuth quadruple “sequential” or “concomitant” therapy should be managed. One potential disadvantage of these therapies is that patients with failed eradication would have limited options for further treatment, because they would already have received 3 different antibiotics: amoxicillin, clarithromycin, and nitroimidazole. However, the recent appearance of levofloxacin may overcome this problem. Thus, Zullo et al. [150] recently performed a pilot study on patients who failed sequential therapy; following 10-day triple therapy with a PPI, levofloxacin, and amoxicillin, *H. pylori* infection was successfully cured in 86% of cases. In another study, Perna et al. [118] prescribed a 10-day triple regimen with a PPI, levofloxacin, and amoxicillin in patients in whom first treatment with either standard 10-day triple or sequential therapy (only 10 patients) had failed. *H. pylori* was eradicated in 73% of cases, although the authors do not provide separate efficacy rates depending on the first (failure) treatment. These data seem to indicate that a triple regimen (PPI-levofloxacin-amoxicillin) is a suitable approach for second-line treatment in patients whose sequential—and probably also concomitant—therapy fails.

## 4. Conclusion

Even with the current most effective treatment regimens, ≥20% of patients will fail to eradicate *H. pylori* infection. This paper seems important at the present time, as therapy for *H. pylori* infection is becoming more and more frequently prescribed. Nowadays, apart from having to know well first-line eradication regimens, we must also be prepared to face treatment failures. Therefore, in designing a treatment strategy we should not only focus on the results of primary therapy alone, but also on the final—overall—eradication rate.

The choice of a “rescue” treatment depends on which treatment is used initially. If a first-line clarithromycin-based regimen was used, a second-line metronidazole-based treatment (such as the quadruple therapy) may be used afterwards, and then a levofloxacin-based combination would be a third-line “rescue” option. Alternatively, it has recently been suggested that levofloxacin-based “rescue” therapy constitutes an encouraging 2nd-line strategy, representing an alternative to quadruple therapy in patients with previous PPI-clarithromycin-amoxicillin failure, with the advantage of efficacy, simplicity, and safety. In this case, quadruple regimen may be reserved as a 3rd-line “rescue” option.

Even after two consecutive failures, several studies have demonstrated that *H. pylori* eradication can finally be achieved in almost all patients if several “rescue” therapies are consecutively given [22, 151]. As a final conclusion, therefore, the attitude in *H. pylori* eradication therapy failure, even after two or more unsuccessful attempts, should be to fight and not to surrender [152].

## Figures and Tables

**Table 1:** Eradication rates with quadruple therapy (proton pump inhibitor, bismuth, tetracycline, and a nitroimidazole) as “rescue” therapy for proton pump inhibitor-clarithromycin-amoxicillin failure.

Author	Number of patients	Duration (days)	Eradication rate (%)
Baena et al. [50]	31	14	90
Bilardi et al. [51]	46	7	37
Elizalde et al. [52]	31	7	87
Choung et al. [53]	56	7	77
Choung et al. [53]	99	14	88
Su et al. [54]	87	7	84
Chung et al. [64]	90	7	82
Chung et al. [64]	101	14	85
Gasbarrini et al. [55]	9	7	88
Gisbert et al. [56]	30	7	57
Gisbert et al. [46]	9	7	78
Gomollón et al. [57]	21	7	95
Lee et al. [58]	20	7	68
Lee et al. [59]	63	7	75
Lee et al. [153]	112	7	64
Lee et al. [153]	115	10	83
Marko et al. [60]	27	7	63
Michopoulos et al. [61]	38	14	76
Navarro-Jarabo et al. [62]	54	7	70
Nista et al. [63]	70	7	63
Nista et al. [63]	70	14	68
Orsi et al. [93]	50	12	88
Perri et al. [154]	45	10	67
Perri et al. [92]	60	7	83
Sicilia et al. [155]	21	10	83
Usta et al. [156]	89	14	67
Uygun et al. [157]	100	14	82
Wong et al. [94]	53	7	91
Wu et al. [158]	47	7	77
Wu et al. [159]	62	7	81

Eradication rates by intention-to-treat analysis when available. *H. pylori* eradication rate (weighted mean) with quadruple therapy: 77%.

## Abbreviations

*H. pylori*: *Helicobacter pylori*
PPI: Proton pump inhibitor.
